# Understanding drivers of domestic public expenditure on reproductive, maternal, neonatal and child health in Peru at district level: an ecological study

**DOI:** 10.1186/s12913-018-3649-x

**Published:** 2018-11-06

**Authors:** Luis Huicho, Patricia Hernandez, Carlos A. Huayanay-Espinoza, Eddy R. Segura, Jessica Niño de Guzman, Gianfranco Flores-Cordova, Maria Rivera-Ch, Howard S. Friedman, Peter Berman

**Affiliations:** 1 Centro de Investigación para el Desarrollo Integral y Sostenible, Universidad Peruana Cayetano Heredia, Av. Honorio Delgado 430, LI33, Lima, Peru; 2 Centro de Investigación en Salud Materna e Infantil and School of Medicine, Universidad Peruana Cayetano Heredia, Lima, Peru; 3 Universidad Nacional Mayor de San Marcos, Lima, Peru; 4 Netherlands Interdisciplinary Demographic Institute, The Hague, The Netherlands; 5 Escuela de Medicina, Universidad Peruana de Ciencias Aplicadas, Lima, Peru; 6 Ministerio de Economía y Finanzas, Lima, Peru; 7 United Nations Population Fund, New York, USA; 8 Department of Global Health and Population, Harvard T.H. Chan School of Public Health, Boston, USA

**Keywords:** Health expenditure, Reproductive health, Maternal health, Neonatal health, Child health

## Abstract

**Background:**

Peru has increased substantially its domestic public expenditure in maternal and child health. Peruvian departments are heterogeneous in contextual and geographic factors, underlining the importance of disaggregated expenditure analysis up to the district level. We aimed to assess possible district level factors influencing public expenditure on reproductive, maternal, neonatal and child health (RMNCH) in Peru.

**Methods:**

We performed an ecological study in 24 departments, with specific RMNCH expenditure indicators as outcomes, and covariates of different hierarchical dimensions as predictors. To account for the influence of variables included in the different dimensions over time and across departments, we chose a stepwise multilevel mixed-effects regression model, with department-year as the unit of analysis.

**Results:**

Public expenditure increased in all departments, particularly for maternal-neonatal and child health activities, with a different pace across departments. The multilevel analysis did not reveal consistently influential factors, except for previous year expenditure on reproductive and maternal-neonatal health. Our findings may be explained by a combination of inertial expenditure, a results-based budgeting approach to increase expenditure efficiency and effectiveness, and by a mixed-effects decentralization process. Sample size, interactions and collinearity cannot be ruled out completely.

**Conclusions:**

Public district-level RMNCH expenditure has increased remarkably in Peru. Evidence on underlying factors influencing such trends warrants further research, most likely through a combination of quantitative and qualitative approaches.

**Electronic supplementary material:**

The online version of this article (10.1186/s12913-018-3649-x) contains supplementary material, which is available to authorized users.

## Background

Investing in health at national and sub-national levels is critical to achieve sustained gains in health and productivity [1]. But investment is not enough and concurrent political will and sustained policies over time are needed to build powerful driving forces in the path to measurable health gains, higher individual and social productivity and sustained country economic growth [1]. Investing specifically in reproductive, maternal, newborn and child health (RMNCH) has shown to be especially important for achieving significant improvement in maternal and child health and nutrition, and for long-term improvement in human and social capital [2].

Focusing only on national level funding trends hides regional disparities, and it does not allow identifying which departments get higher funding and which are lagging behind and therefore, it does not reveal whether funding trends are in line with trends in coverage and impact indicators. The scant literature on subnational financing trends and determinants in low and middle-income countries suggests that greater subnational health expenditure reflects a higher degree of democratization and local autonomy and a better understanding by local policy makers of the local context and needs [3].

Peru has achieved impressive progress in RMNCH over the last two decades [4–6]. It has already reached the Millennium Development 4 (MDG4) target for under-five mortality reduction and although it did not reach the MDG5, it reduced substantially its maternal mortality ratio to 89 per 100,000 live births in 2013 [5, 6]. Peru has also made significant reductions in neonatal mortality and particularly early neonatal mortality [5, 7]. More recently, after a long period of stagnation, it has been able to reduce under-five stunting to levels below 15% [8]. It is likely that such improvements would not have been possible without substantial financial investment in the design, implementation, monitoring and evaluation of RMNCH specific activities, although no systematic attempts have been made to relate the progress in health with funding trends.

Domestic financial expenditure is considered a key component in country efforts to improve health [9], and Peru is a remarkable example of substantial progress achieved in RMNCH while relying mostly on its own financial resources [10]. The analysis of sub-national public expenditure in health and RMNCH is particularly important to identify inequities in the allocation of resources relative to need. Available expenditure levels may impact on the intensity of programme implementation, affecting the coverage of the service within a population, so as to identify fast and slow progressing regions, and to explore possible success factors as well as relevant bottlenecks.

Countdown to 2015, a global initiative to monitor country level progress in maternal and child health, has been tracking the external expenditure for RMNCH activities at the national level among priority countries [4]. However, sub-national level trends and analyses of RMNCH expenditure have not been included up to now, due to the absence or very limited data availability.

We performed this sub-national financial flow analysis study as part of a Countdown to 2015 series of country case studies that aim to describe trends in public RMNCH expenditure. The current study aims to estimate levels of domestic public RMNCH expenditure at the department level in Peru, and to explore associations between various categories of potential driving factors as determinants of expenditure variation and specific domestic RMNCH expenditure outcomes, for the period 2000 to 2012. The level of domestic expenditure at national level has been explored in a separate paper [10].

## Methods

### Study setting

Peru is an upper-middle income country geographically divided in three natural regions, namely Coast, Andes and Amazon. Most of the rural and poorest populations are concentrated in the latter two regions. Peru is administratively divided into 24 departments. There is wide variation in the economic, geographical and cultural characteristics of these departments. Lima, Arequipa, Moquegua and La Libertad are examples of large, predominantly coastal departments, with high concentration of economic, political and administrative activities. Ayacucho, Huancavelica and Pasco are representative of Andean departments, while Madre de Dios, Iquitos and Ucayali are predominantly Amazon departments. These last two groups of departments concentrate the largest proportion of the country’s rural (88.8%) and poor populations (65.8%), and are characterized by a lower economic development.

A regional government holds political, administrative and economic responsibilities for each department, with a high degree of autonomy. However, final decision on funding allocation to departments is made at central government level, upon annual demonstration of accomplishment of programmatic goals. In addition, departments are allowed to generate their own revenues by providing semi-subsidized health services or charging those not eligible for the subsidized services. The technical capacity of departments to design and implement successfully sound development projects and quality health services is still limited, and this drawback is compounded by low levels of governance and accountability, and by the shortage of trained and motivated health personnel in the areas where they are most needed [11]. Thus, decentralization has brought mixed results and remains a pending challenge in the accomplishment of an effective subnational development and in the effective provision of essential public services, such as health care [11].

Peru has a mixed health system in which the public and private sectors coexist. The first includes the Ministry of Health and the Social Security (ESSALUD), along with the Police and Army sectors [11]. The Ministry of Health provides health care through the Comprehensive Health Care Insurance (SIS), which is mainly based on a subsidized scheme and it is focused on the poor. ESSALUD is funded by payroll taxes from the employed workforce. The private sector comprises a not-for-profit segment that mainly includes non-governmental organizations, and a for-profit segment that includes private insurance companies and service providers. As part of the decentralization process, the Ministry of Health has transferred to the regional governments the responsibility of providing health services, although the Ministry of Economy and Finances, at the central level, retains the funding allocation responsibility [12].

### Conceptual framework

**Fig. 1:**
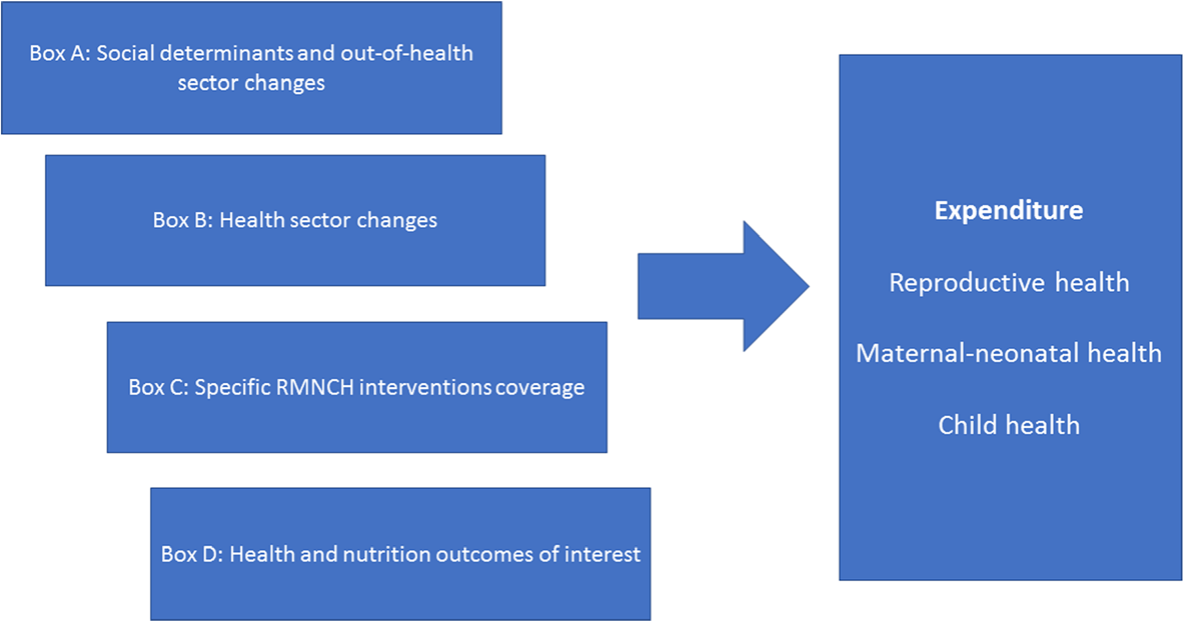
Conceptual framework of hierarchical variables, from distal to proximal determinants

We adapted a conceptual framework initially prepared for the extended country case study [10], which encompasses various hierarchical levels of variables that may lead to changes in health and nutrition indicators, from distal to proximal determinants, including socioeconomic factors and out-of-health sector changes, health sector changes (including public RMNCH expenditure), coverage of RMNCH interventions, and health impact indicators (nutrition and mortality). As we point out, the current study will explore the influence of diverse predictor variables on the domestic expenditure levels, our adapted framework included four levels (boxes) of variables, as follows (Fig. 1). Box A includes social determinants and out-of-health sector changes: Gross Domestic Product (GDP) per capita in constant 2012 US$, Gini coefficient for income, percentage of families living below the poverty line, percentage of families with at least one unmet basic need, percentage of urban population, median years of schooling among women 15 years and older, total fertility rate and percentage of rural families that are beneficiaries of conditional cash transfer program JUNTOS. Box B involves health sector changes: utilization of the SIS in terms of number of annual under-five outpatient preventative and clinical attendances per total under-five population, density of doctors, nurses and midwives per 10,000 population, and expenditure on RMNCH health on previous year in constant 2012 US$. Box C comprises specific RMNCH interventions coverage (percentage of women with family planning needs satisfied, percentage of pregnant women with at least four antenatal care visits, percentage of live births attended by skilled health personnel, percentage of infants who received three doses of DPT vaccine, percentage of infants immunized with measles vaccine, percentage of under-five children with suspected pneumonia taken to an appropriate health provider, percentage of under-five children with diarrhoea receiving oral rehydration salts and continued feeding). Box D includes health and nutrition outcomes of interest: under-five stunting prevalence (percentage of children under-five years whose height or length for age is below two standard deviations from the median, according to the World Health Organization growth standards) [13], estimated by pooling births and deaths by calendar years for all children born to women interviewed in 10 demographic and health surveys, DHS [14]. Mortality rates were estimated for four three-year periods: 2001–2003, 2004–2006, 2007–2009, 2010–2012).

For performing the main analyses of this paper, we used indicators of public RMNCH expenditure as the specific outcomes.

Building upon the conceptual framework, we hypothesized that, at departmental level, the outcomes of interest (expenditure on reproductive health, expenditure on maternal-neonatal health, and expenditure on child health) would be influenced by perceived need of departments by those responsible of budget allocation and Execution. Departments with worsening, stagnant or limited improvement of social determinants and absence or limited out-of-health sector changes such as access to water at departmental level would be perceived as in more need. Likewise, the progress achieved in the delivery of services by the health sector, by the coverage level achieved for RMNCH interventions, and by the progress achieved in terms of maternal, neonatal and child mortality and in under-five stunting prevalence would influence the need perception of policy makers and managers and therefore the expenditure efforts made to improve the situation. We anticipated that the departments in most need, those with the lowest coverage for RMNCH interventions and those with the worst figures for maternal, neonatal and child mortality and under-five stunting prevalence would show the highest progress in public RMNCH expenditure, reflecting a preferential status in the financial allocation process by the central government level and the greatest expenditure efforts at local level, in line with a concerted national agreement set up in the early 2000s that aimed at prioritizing the poorest regions of the country, but also at emphasising local technical and managerial capacities to improve public RMNCH expenditure efficiency [15].

### Data sources

Data on domestic public expenditure on RMNCH were obtained from the Ministry of Economy and Finances of Peru website [16]. The information is collected from all the Peru departments routinely, as part of fiscal decentralization regulations in place, and it is reported quarterly to the Ministry of Economy and Finances (MoF). The reported information is grouped by expenditure categories (regular resources, resources directly collected, resources from official credit operations, donations and transfers, and specified resources), by time period (quarter) and by department (region). Then a quality control process is performed, and the information is systematized and uploaded in the online information system of the MoF, known as The Financial Management Information System, which is publicly available, in compliance of the transparency government policy [16].

Information on GDP, percentage of urban population, and population under five years of age were obtained from the National Institute of Statistics and Computing [17, 18]. Poverty line and unmet basic needs data were derived from the national Demographic and Health Surveys (DHS) [8]. The Gini coefficients for income, access to water, women’s schooling and fertility were obtained from DHS [8]. Information on coverage of the conditional cash transfer program JUNTOS was derived from the JUNTOS website [19]. The DHS are conducted following internationally agreed methodological standards. Moreover, we used a DHS-based dataset rigorously standardised by the International Center for Equity in Health, a widely recognized scientific center that is in charge of providing the DHA-based data for the Countdown to 2030 monitoring tasks at country and subnational levels.

Data on SIS were obtained from its official website [20]. Density of doctors, nurses and midwives was obtained on data from the Human Resources Department of the Ministry of Health website [21]. Coverage of RMNCH interventions and prevalence of stunting were derived from the DHS [14], and neonatal mortality rate and under-five mortality rate were estimated from multiple DHS, as described above. For interventions like JUNTOS and SIS, implementation characteristics including definition and target groups have been described elsewhere [10].

The institutional websites used for our model belong to official links of diverse government sectors, and are of public domain. Such publicly available information sources allow the readers judging by themselves the quality and reliability of the provided information. We acknowledge however, that language may be an issue for international readers, as the webpages are in Spanish.

### Missing data and imputation

The vast majority of study variables had no missing values. We had incomplete data for density of doctors, nurses and midwives for years 2000, 2005, 2008 and 2011, due to lack of consistently and reliably collated information from the official source. As for stunting prevalence, the annual available DHS datasets had missing data for years 2004 and 2006. Two different imputation methods, regression-based and tree-based imputation were employed to explore the sensitivity of the results to the imputation method [22, 23]. For regression-based imputation, simple linear regressions of the variable of interest against time (year) were run [22]. SAS Enterprise Miner 4.3 was used to develop tree-based imputations (with surrogates) for missing class and interval variables, and imputed indicators were not developed as part of this imputation method [23]. The regression-based and tree-based imputed variables were consistent, and thus only one of them (regression-based) was used in the ecological analyses.

### Allocating expenditures to RMNCH activities

We accessed to financial data on RMNCH from the Ministry of Economy and Finances, detailed by specific activities included within nutritional, maternal-neonatal and child health programmes with defined target groups and objectives [24]. Expenditures were classified independently by three researchers (LH, ERS, JNG) into three groups: reproductive health (defined as expenditure on activities related to contraception, family planning, HIV and other sexually transmitted diseases), maternal and newborn health (defined as expenditure on antenatal care, birth attendance and postnatal care activities for mothers and newborns), and child health (defined as expenditure on preventative and curative activities targeted to under-five children excluding the neonatal period). Discrepancies were resolved by consensus. The grouping process resulted in the following data periods: 2004–2012 for reproductive health, and 2000–2012 for maternal-neonatal and child health.

### Data analysis

We estimated expenditure data in constant 2012 US$, which take inflation into account. We transformed original expenditure data available in local currency (PEN), to constant 2012 US$ by using currency exchange rates for each year, and then dividing the results by national 2012 deflator from the World Development Indicators database [25].

We plotted expenditure annual trends in reproductive health per woman of reproductive age, in maternal and newborn health per pregnant woman, and in child health per child under five by department for the corresponding period. The departments were ranked by their average annual change in per capita expenditure for each outcome over the study period, which was estimated through linear regression of the corresponding outcome against year.

We additionally compared baseline and most recent year reproductive, maternal-neonatal or child health expenditures at departmental level, expressed in constant 2012 US$.

We ran departmental level bivariate correlations between expenditure indicators and social determinants, out-of-health and within-health sector changes, coverage of RMNCH interventions, and mortality over the study period.

To assess the independent influence of the variables included in our conceptual framework on RMNCH outcomes over time and across departments [26], we used a stepwise multilevel mixed-effects regression model, where the unit of analysis is department-year. The model takes into account the fixed effects of the variables in the different dimensions and the fixed effects of time, while the random effects take into account the variability between departments [27].

We considered a one-year time lag to allow a reasonable period of time between predictors and outcomes, both for bivariate correlations and for ecological multilevel analyses, considering that the predictors must temporally precede the outcomes, so the analysis is causally one-way oriented.

For the multilevel analyses, we used a parsimonious model. That is, the model variables within each box A were selected from our general conceptual framework, according to the available evidence about their influence on the particular outcome involved. Thus the covariates initially selected within each box for running the crude regressions may vary to some extent for each outcome (expenditure on reproductive health, expenditure on maternal-neonatal health or expenditure on child health). Also, variables with similar constructs were excluded a priori, to avoid multicollinearity. For example, instead of including both variables related to the percentage of households living below the poverty line and the percentage living below the extreme poverty line, we retained only poverty line.

Crude and adjusted multilevel regressions were run separately for each outcome. For each box (starting with box A) of our conceptual model, we first ran crude mixed-effects linear regressions, with the outcome variable and one predictor at a time. We selected variables with *p* ≤ 0.20 [28], to run an adjusted multi-level mixed-effects linear regression, where time was a locked term, that is a variable included in the model irrespective of the selection criteria. Then, based on the results, we performed a backward stepwise selection, by excluding in a sequential way, one at a time, variables with *p*-values higher than 0.20, starting with those with the highest p-value. We obtained in this way a final model (for each box), with variables that had to be incorporated in the final models of the following boxes. Then we repeated the same crude regression analysis of the outcome with each predictor in box B, as well as the backward stepwise selection for variables with *p* ≤ 0.20. The final selected variables in box B and the final selected variables from box A were run all together in a new multivariate model. Afterwards, a new backward stepwise selection was performed, to obtain the final model for box A plus B. Similarly, variables of this final model were kept for incorporation in the final models of the following boxes, as far as they had p ≤ 0.20.

We repeated these steps with box C and box D including the selected variables from previous boxes. In the final model, we considered as statistically significant those variables with *p* < 0.05.

## Results

### Time trends for departmental RMNCH expenditure

**Fig. 2:**
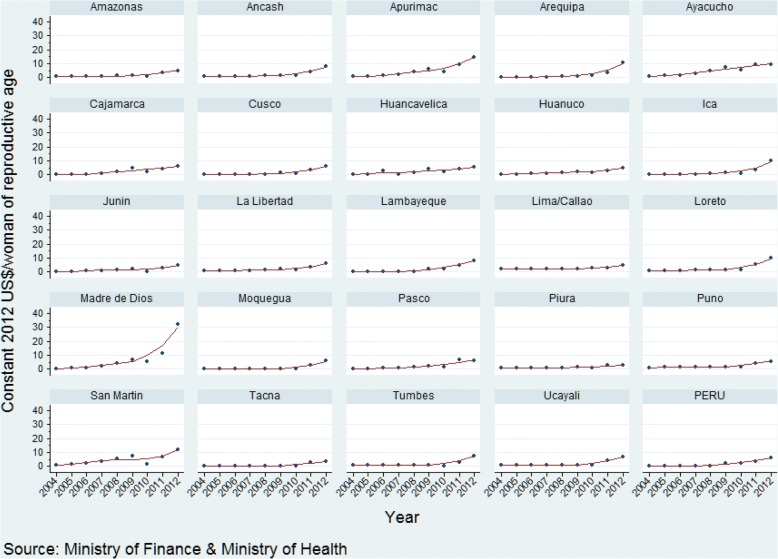
Per capita expenditure on reproductive health activities (constant 2012 US$) at departmental level, Peru: 2004–2012

**Fig. 3:**
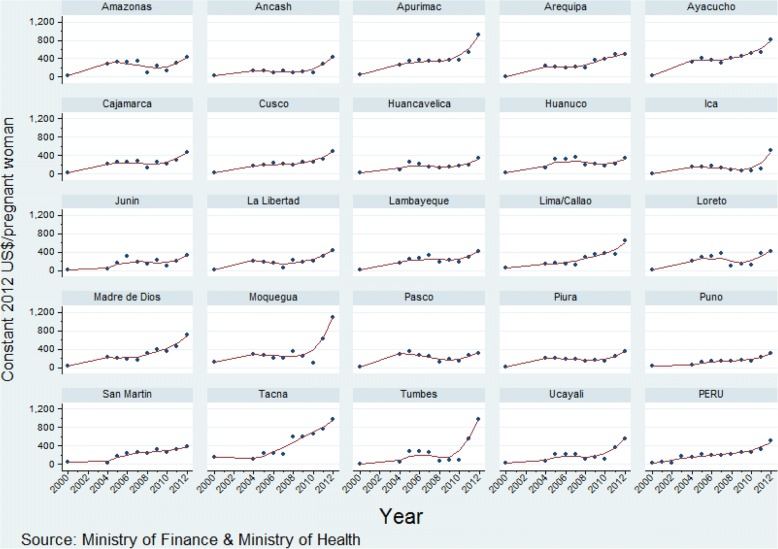
Per capita expenditure on maternal-neonatal health activities (constant 2012 US$) at departmental level, Peru: 2000–2012

**Fig. 4:**
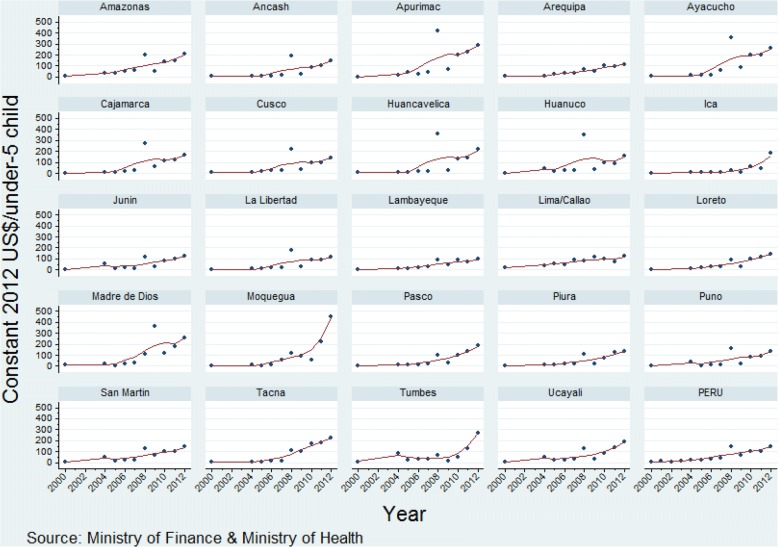
Per capita expenditure on child health activities (constant 2012 US$) at departmental level, Peru: 2000–2012

There has been a consistent increase in maternal-neonatal health expenditure per pregnant woman and for child health expenditure per child under five over the study period, with a less pronounced increase in expenditure on reproductive health per woman of reproductive age across departments (Figs. 2, 3, 4; Additional files 1, 2, 3). The increase in expenditure in all cases was steeper from 2008. Over time visual differences between departments are evident for each of the three categories of expenditure. Detailed annual per capita RMNCH expenditure information, at departmental level, is provided in Additional files 1, 2, 3 [16].

Ayacucho and Apurimac departments increased the most for all three indicators (Table 1). The departments that increased annual expenditure on reproductive health per woman of reproductive age the most were Arequipa, San Martín, Ayacucho, Apurímac and Madre de Dios (average annual increase range, US$ 0.9 to US$ 2.9 per woman of reproductive age), while the departments increasing expenditure the least were Lima, Piura, Tacna, Amazonas, and Junin (range, US$ 0.2 to US$ 0.4). As for expenditure on maternal and neonatal health, the departments with the highest average annual increase were Ayacucho, Moquegua, Apurimac, Tumbes, and Tacna (annual increase range, US$ 49.5 to US$ 73.9 per pregnant woman), while those with the lowest average annual increase were Pasco, Huánuco, Amazonas, Huancavelica, and Piura (range, US$ 9.4 to US$ 16.5). The departments with the greatest annual increase in child health expenditure per child under five were Tacna, Madre de Dios, Ayacucho, Apurimac, and Moquegua (annual increase range, US$ 20.7 to 28.1 per under-five child), while the departments with the smallest annual increase in expenditure were Lima, Lambayeque, Junin, Arequipa, and Puno (range, US$ 8.3 to 10.2).

**Table 1:** Ranking of departments by annual increase of per capita expenditure

Department	Per capita expenditure on reproductive health (constant 2012 US$/woman of reproductive age), 2004–2012		Per capita expenditure on maternal-neonatal health (constant 2012 US$/pregnant woman), 2000–2012		Per capita expenditure on child health (constant 2012 US$/under-five child), 2000–2012	
	Beta	SE	Beta	SE	Beta	SE
Amazonas	0.38	0.14	15.37	11.51	16.03	4.47
Ancash	0.65	0.20	22.14	8.14	12.42	4.99
Apurimac	1.50	0.28	51.13	12.07	25.93	10.41
Arequipa	0.91	0.32	38.59	5.25	9.64	1.62
Ayacucho	1.19	0.13	49.50	8.25	24.40	8.56
Cajamarca	0.75	0.14	23.02	7.72	15.51	6.76
Cusco	0.57	0.16	29.65	5.33	12.24	5.46
Huancavelica	0.58	0.15	15.61	6.37	18.47	9.66
Huanuco	0.47	0.09	15.01	9.17	12.66	9.18
Ica	0.83	0.29	18.06	11.39	10.33	3.93
Junin	0.40	0.12	18.22	7.95	9.54	3.13
La Libertad	0.50	0.14	25.71	7.26	10.69	4.44
Lambayeque	0.88	0.19	22.23	6.98	8.66	1.86
Lima	0.23	0.08	41.77	8.44	8.31	1.59
Loreto	0.82	0.27	19.15	11.67	11.78	2.61
Madre de Dios	2.87	0.86	47.02	8.90	24.00	8.37
Moquegua	0.51	0.19	50.76	23.56	28.08	9.27
Pasco	0.75	0.17	9.43	9.67	14.56	3.56
Piura	0.27	0.07	16.51	6.22	11.03	3.12
Puno	0.44	0.14	18.57	3.52	10.25	4.32
San Martin	0.98	0.32	27.75	4.67	11.11	2.90
Tacna	0.39	0.12	73.86	13.84	20.70	4.03
Tumbes	0.53	0.24	51.22	23.03	13.39	6.16
Ucayali	0.54	0.21	30.40	10.68	13.14	3.95

**Fig. 5:**
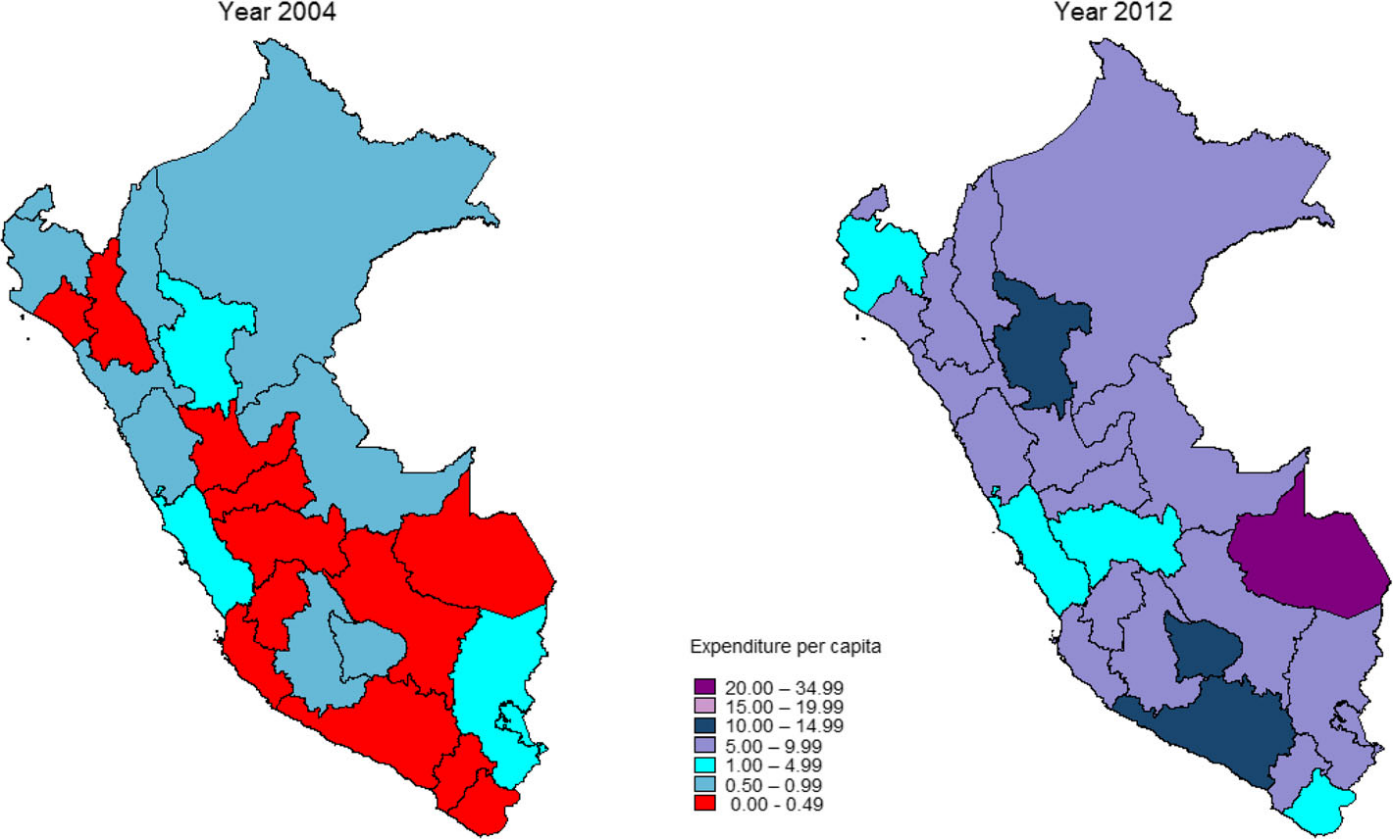
Expenditure per capita on reproductive health activities (constant 2012 US$), Peru: 2004 and 2012. Data source: Ministry of Finance, Sistema de Administración Financiera (SIAF) [http://apps5.mineco.gob.pe/transparencia/Navegador/default.aspx] (Open access)

**Fig. 6:**
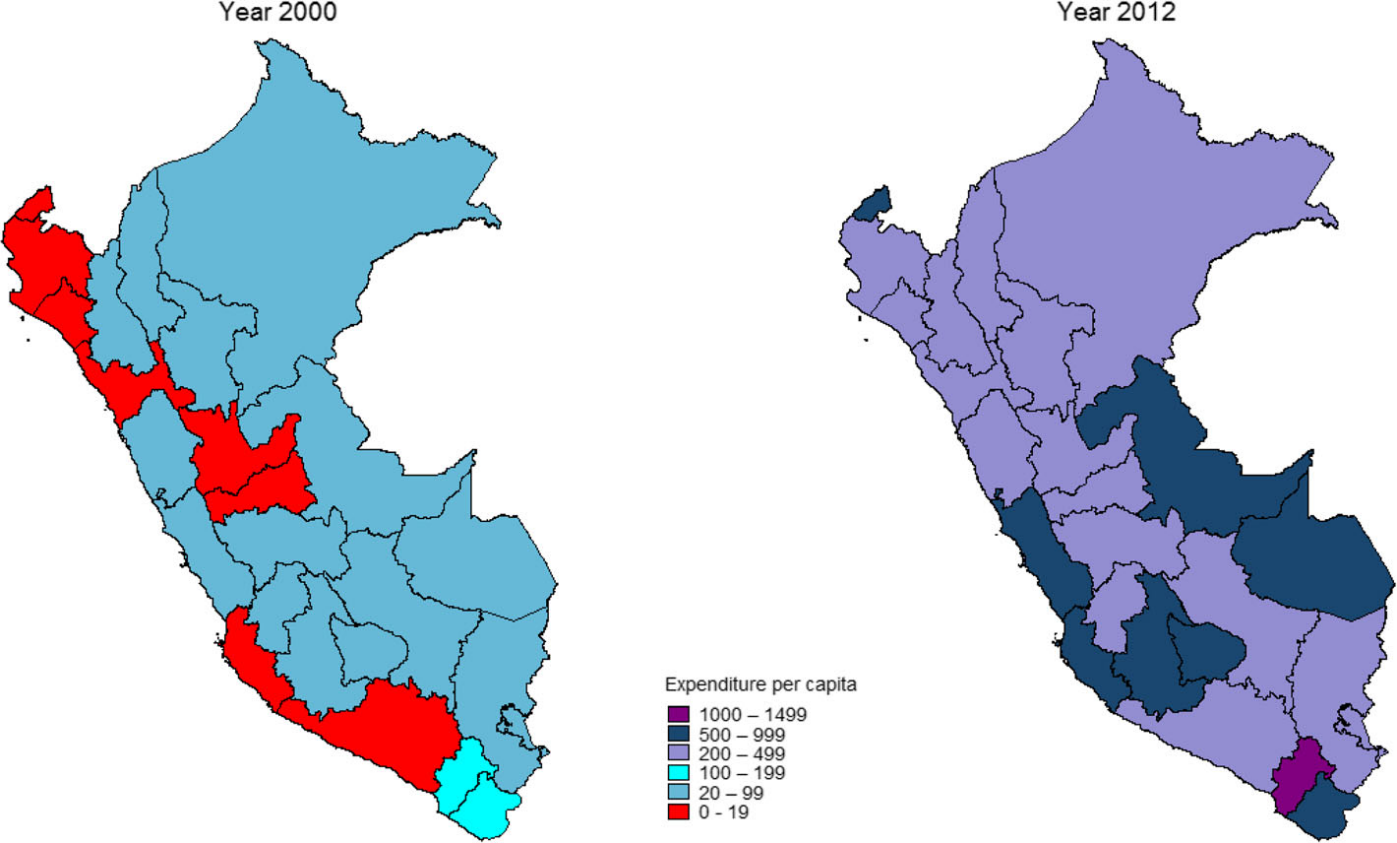
Expenditure per capita on maternal and neonatal health activities (constant 2012 US$), Peru: 2000 and 2012. Data source: Ministry of Finance, Sistema de Administración Financiera (SIAF) [http://apps5.mineco.gob.pe/transparencia/Navegador/default.aspx] (Open access)

**Fig. 7:**
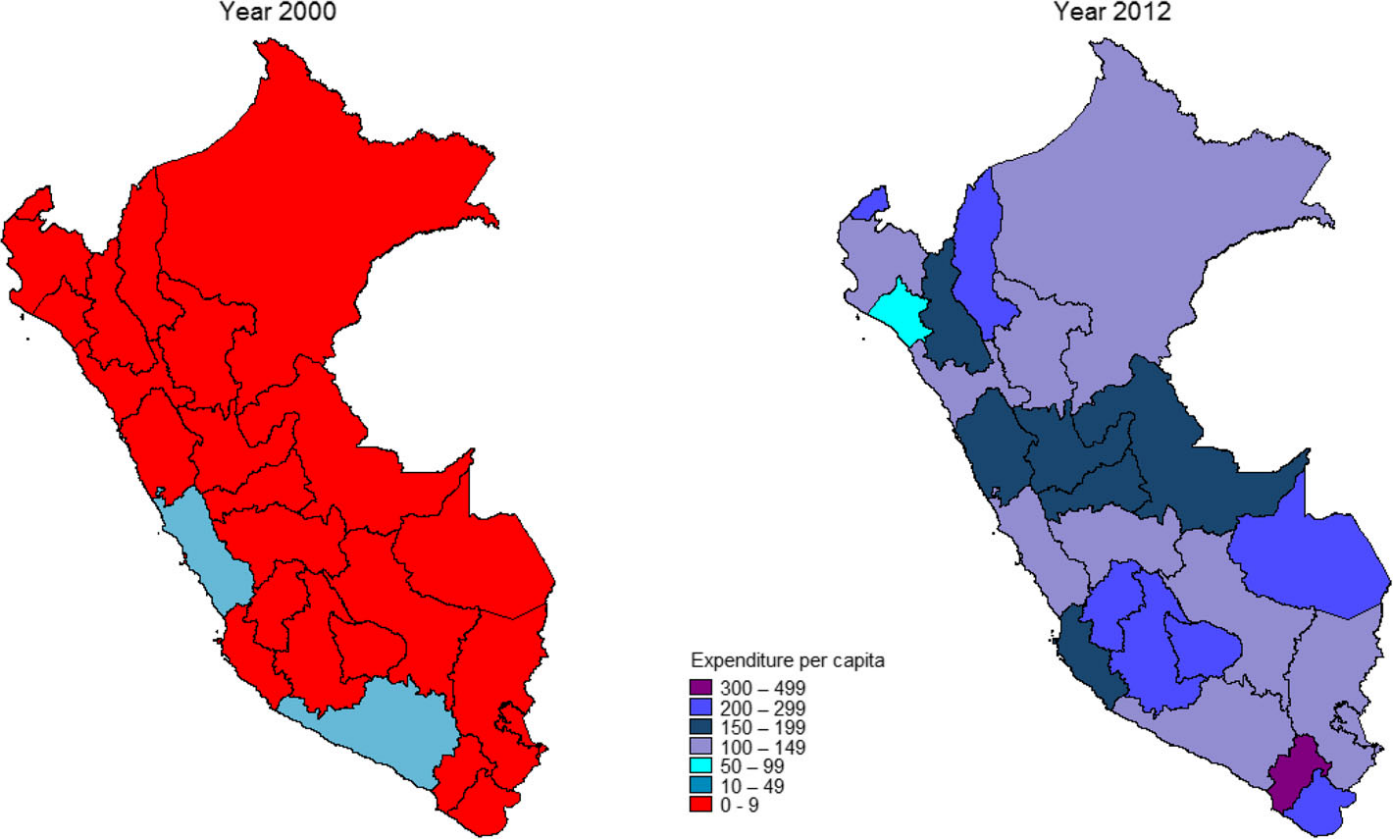
Expenditure per capita on child health activities (constant 2012 US$), Peru: 2000 and 2012. Source: Data source: Ministry of Finance, Sistema de Administración Financiera (SIAF) [http://apps5.mineco.gob.pe/transparencia/Navegador/default.aspx] (Open access)

Figures 5, 6, 7 show the change in levels of expenditure between baseline and most recent year for each of the expenditure indicators. Twelve departments showed a per capita expenditure on reproductive health of less than 0.5 US$ per woman of reproductive age in 2004, while in 2012 none spent less than that amount (Fig. 4). Conversely, while in 2004 not a single department spent more than US$ 5 per woman of reproductive age, in 2012 there were 20 departments that spent more than this amount (Fig. 5).

As for per capita expenditure on maternal-neonatal health, 22 departments spent less than US$ 20 per pregnant woman in 2000, while in 2012 none spent less than that figure (Fig. 5). By contrast, in 2000 not a single department spent more than US$ 199, and in 2012 all 24 departments spent US$ 200 or more (Fig. 6).

Finally, for per capita expenditure on child health, 22 departments spent less than US$ 9 per child and only one (Lima) spent US$ 15.9 in year 2000. In year 2012, all except one department (Lambayeque) spent US$ 100 or more (Fig. 7).

### Bivariate correlations

Table 2 shows bivariate correlations between per capita expenditure outcomes (either reproductive, maternal-neonatal or child health) and contextual, out-of-health sector, health sector, coverage and mortality indicators at departmental level. The Gini coefficient and the share of population below the poverty line showed negative significant correlations with reproductive, maternal- neonatal and child health expenditures per capita. Unmet basic needs also showed negative significant correlations with maternal-neonatal and child health (although weaker than Gini and poverty line). The conditional cash transfer programme JUNTOS that targets the poorest segments of the population, had positive significant correlations with reproductive and child health expenditure, but comparatively low and not significant correlation with maternal-neonatal expenditure.

**Table 2:** Bivariate correlations of expenditure outcomes with covariates from different dimensions (one year time-lag for outcomes)

Box	Covariate	Per capita expenditure on reproductive health		Per capita expenditure on maternal-neonatal health		Per capita expenditure on child health	
		Coefficient	*p*	Coefficient	*p*	Coefficient	*p*
Box A	GDP per capita (constant 2012 US$)	0.081	0.263	0.277	0.000	0.163	0.024
	Gini for income (%)	−0.390	0.000	− 0.438	0.000	−0.334	0.000
	Poverty line (%)	−0.277	0.000	−0.321	0.000	− 0.241	0.001
	Unmet basic needs (%)	−0.116	0.108	−0.273	0.000	−0.169	0.019
	Urbanization (%)	−0.024	0.737	0.109	0.132	−0.102	0.160
	Median years of schooling, women	−0.018	0.809	0.117	0.107	−0.056	0.444
	Total fertility rate	0.078	0.282	−0.098	0.178	0.048	0.511
	Cash Transfer Program coverage (% of rural families beneficiaries of JUNTOS)	0.255	0.000	0.132	0.067	0.426	0.000
Box B	Health insurance system (SIS) utilization (attendances/under-five child)	0.066	0.362	−0.018	0.804	0.079	0.275
	Density of doctors, nurses and midwives (per 10,000 pop)	0.365	0.000	0.498	0.000	0.545	0.000
	Per capita expenditure on reproductive health, previous year (constant 2012 US$/woman of reproductive age)	0.829	0.000	0.533	0.000	0.550	0.000
	Per capita expenditure on maternal-neonatal, previous year health (constant 2012 US$/pregnant woman)	0.461	0.000	0.782	0.000	0.546	0.000
	Per capita expenditure on child health, previous year (constant 2012 US$/under-5 child)	0.524	0.000	0.490	0.000	0.379	0.000
Box C	Family planning needs satisfied (%)	0.042	0.559	0.107	0.140	0.119	0.101
	Antenatal care visits (%)	0.204	0.005	0.411	0.263	0.284	0.000
	Skill birth attendance (%)	0.202	0.005	0.386	0.299	0.230	0.001
	DPT vaccine coverage (%)	−0.075	0.300	−0.016	−0.034	− 0.140	0.052
	Measles vaccine coverage (%)	0.077	0.291	0.151	0.145	0.097	0.183
	Careseeking for pneumonia (%)	−0.067	0.355	− 0.005	− 0.104	−0.133	0.066
	ORT (%)	0.111	0.126	0.229	0.062	0.056	0.439
	Composite coverage index (%)	0.090	0.216	0.286	0.157	0.089	0.220
Box D	Stunting (%)	−0.088	0.227	−0.250	0.001	−0.054	0.460
	Neonatal mortality rate (per 1000 live births)^a^	− 0.104	0.153	−0.264	0.000	−0.301	0.000
	Under-5 mortality rate (per 1000 live births)^a^	− 0.177	0.014	−0.346	0.000	−0.297	0.000

^a^Based on multiple DHS (birth cohorts). DPT: Diphtheria, Pertussis and Tetanus. ORT: oral rehydration therapy and continued feeding

As for health sector changes, expenditure on previous year and density of doctors, nurses and midwives had strong and positive correlations with each reproductive, maternal-neonatal and child health expenditures per capita (Table 2).

As for specific RMNCH interventions, coverage indicators across the continuum of care showed in general, weak correlations with each of the three expenditure outcomes (Table 2). Coefficients varied from 0.04 (for correlations between family planning needs satisfied with expenditure on reproductive health), to 0.4 (for correlation between antenatal care with expenditure on maternal-neonatal health).

With regard to impact indicators, while there was no significant correlation between stunting and reproductive and child health expenditures per capita, it showed a negative significant correlation with maternal-neonatal expenditure per pregnant woman (correlation coefficient − 0.25, *p* = 0.001) (Table 2). Neonatal mortality had a significant negative correlation with expenditure on maternal-neonatal health per pregnant woman (correlation coefficient − 0.26, *p* < 0.0001) and with expenditure on child health per child (correlation coefficient − 0.30, *p* < 0.0001) (Table 2). Under-five mortality rate had a significant negative correlation with expenditure on maternal-neonatal health (correlation coefficient − 0.35, *p* < 0.0001), with expenditure on child health (correlation coefficient − 0.30, *p* < 0.0001) and with reproductive health expenditure (correlation coefficient − 0.18, *p* = 0.014), although this last correlation was weaker (Table 2).

There was a lack of consistent association between annual change of coverage and annual change of impact indicators with annual change of specific RMNCH expenditure, where each dot is one department (Additional file 4).

### Ecological multilevel analyses at departmental level

#### Public expenditure on reproductive health

Table 3 shows the results of multilevel analysis with reproductive health expenditure per woman of reproductive age as the outcome.

**Table 3:** Multilevel linear models for expenditure on reproductive health (time lag of 1 year)^a^

Box	variables	Time-adjusted regression coefficient	95% CI	*p*	Time and confounder-adjusted^a^ regression coefficient	95% CI	*p*
	Time (year)	0.87	0.72–1.02	< 0.001	0.19	0.04–0.34	0.013
Box A	GDP per capita (constant 2012 US$)	0.00	0.00–0.00	0.259	–	–	–
	Gini for income (%)	−0.08	− 0.18 - 0.01	0.078	− 0.08	− 0.13 - -0.03	0.003
	Poverty line (%)	0.02	−0.02 - 0.05	0.335	–	–	–
	Unmet basic needs (%)	0.01	−0.03 - 0.05	0.614	–	–	–
	Urbanization (%)	−0.01	−0.04 - 0.02	0.475	–	–	–
	Cash Transfer Program coverage (%)	−0.01	− 0.04 - 0.02	0.581			
	Median years of schooling, women	−0.02	−0.25 - 0.22	0.898			
	Total fertility rate	0.31	−0.43 - 1.05	0.412	–	–	–
Box B	Density of doctor, nurses and midwives (per 10,000 population)	0.06	−0.05 - 0.17	0.29	–	–	–
	Expenditure on reproductive health activities, previous year (constant 2012 US$/woman of reproductive age)	1.29	1.11–1.47	< 0.001	1.28	1.11–1.46	< 0.001
Box C	Family planning needs satisfied (%)	−0.04	−0.15 - 0.07	0.502	–	–	–
Box D	Neonatal mortality rate (per 1000 live births)	0.05	−0.05 - 0.15	0.325	–	–	–

^a^Units of analyses are 192 (24 departments × 8 years). Variables in each group are adjusted for all other variables in the same group or above

The Gini coefficient (*p* < 0.1) and previous year per capita expenditure on reproductive health (*p* < 0.001) were the only variables kept for inclusion in the time- and confounder-adjusted final model. For Gini the coefficient was negative, while it was positive for previous year expenditure. Higher income inequality was associated with lower expenditure on reproductive health, while higher previous year expenditure on reproductive health was associated with higher expenditure on reproductive health over the study period. Differences across departments were not significant in the final adjusted model.

#### Public expenditure on maternal and neonatal health

For time-adjusted coefficients, Gini coefficient, conditional cash transfer JUNTOS, density of health professionals, health insurance system (SIS), and previous year maternal and neonatal health expenditure were significantly associated with maternal and neonatal expenditure over time (Table 4). Expenditure was higher in departments with lower income inequality (lower Gini coefficient), lower coverage of conditional cash transfer programme JUNTOS and lower levels of under-five health services utilization provided by the health insurance system (SIS).

**Table 4:** Multilevel linear models for expenditure on maternal-neonatal health (time lag of 1 year)^a^

Box	Variables	Time-adjusted regression coefficient	95% CI	*p*	Time and confounder-adjusted* regression coefficient	95% CI	*p*
Box A	Time (year)	33.58	25.35–41.81	< 0.001	17.1	8.48–25.72	< 0.001
	GDP per capita (constant 2012 US$)	0.02	−0.02 - 0.05	0.278	–	–	–
	Gini for income (%)	−8.78	−13.99 - -3.57	0.001	−2.36	−5.81 - 1.10	0.181
	Poverty line (%)	0.11	−1.86 - 2.08	0.914	–	–	–
	Unmet basic needs (%)	−0.51	−2.65 - 1.63	0.642	–	–	–
	Urbanization (%)	0.42	−1.35 - 2.18	0.644	–	–	–
	Cash Transfer Program coverage (%)	−2.52	−4.12 - -0.92	0.002	−0.65	−1.61 - 0.30	0.182
	Median years of schooling, women	4.9	−9.18 - 18.98	0.495	–	–	–
	Total fertility rate (%)	−1.35	−44.88 - 42.19	0.952	–	–	–
Box B	Density of doctor, nurses and midwives (per 10,000 population)	9.00	3.19–14.80	0.002	3.47	−0.56 - 7.50	0.091
	Health insurance system, SIS (Attendances/under-5 child)	−17.98	−35.79 - -0.17	0.048	–	–	–
	Expenditure on maternal and neonatal health activities, previous year (constant 2012 US$ per pregnant woman)	0.93	0.80–1.06	< 0.001	0.86	0.72–1.00	< 0.001
Box C	Antenatal care visits (%)	−4.35	−8.73 - 0.04	0.052	–	–	–
	Skill birth attendance (%)	−0.43	−2.28 - 1.42	0.649	–	–	–
Box D	Neonatal mortality rate (%)	−0.59	−6.23 - 5.06	0.838	–	–	–

^a^Units of analyses are 192 (24 departments × 8 years). Variables in each group are adjusted for all other variables in the same group or above

Time- and confounder-adjusted coefficients showed that only Gini and previous year expenditure on reproductive health were statistically significant and remained in the same direction (Table 4).

In the final multilevel model, the random-effects component did not show significant variation across departments.

#### Public child health expenditure

Time-adjusted coefficients showed that higher coverage of conditional cash transfer JUNTOS, higher density of health professionals, higher SIS utilization by under-five children and higher coverage of measles vaccination were associated with higher expenditure on child health over time (Table 5). Conversely, higher urbanization was associated with lower child health expenditure (Table 5).

**Table 5:** Multilevel linear models for expenditure on child health (time lag of 1 year)^a^

Box	Variables	Time-adjusted regression coefficient	95% CI	*p*	Time and confounder-adjusted^a^ regression coefficient	95% CI	*p*
Box A	Time (year)	21.78	17.72–25.83	< 0.001	16.8	11.89–21.71	< 0.001
	GDP per capita (constant 2012 US$)	0.00	−0.02 - 0.01	0.499	–	–	–
	Gini for income (%)	−0.51	−2.62 - 1.61	0.639	–	–	–
	Poverty line (%)	0.46	−0.18 - 1.11	0.161	–	–	–
	Unmet basic needs (%)	0.14	−0.64 - 0.91	0.728	–	–	–
	Urbanization (%)	−0.64	− 1.15 - -0.13	0.014	−0.5	−1.01 - 0.02	0.059
	Cash Transfer Program coverage (%))	0.99	0.40–1.57	0.001	0.7	0.04–1.35	0.036
	Median years of schooling, women	−3.75	−8.22 - 0.73	0.101	–	–	–
	Density of doctor, nurses and midwives (per 10,000 population)	5.74	3.74–7.74	< 0.001	6.35	4.40–8.31	0
	Health insurance system, SIS (Attendances/under-5 child)	7.08	0.90–13.26	0.025	–	–	–
	Expenditure on under-5 health activities (constant 2012 US$ per u5 child)	−0.10	−0.25 - 0.05	0.182	−0.19	−0.33 - -0.05	0.009
Box C	DPT vaccine coverage (%)	−0.09	−1.15 - 0.96	0.860	–	–	–
	Measles vaccine coverage (%)	1.22	0.08–2.37	0.036	0.69	−0.30 - 1.67	0.171
	Care seeking for pneumonia (%)	−0.50	−1.11 - 0.12	0.113	−0.63	−1.18 - -0.09	0.022
	ORT (%)	−0.28	−0.98 - 0.42	0.432	–	–	–
Box D	Under-5 mortality rate (per 1000 live births)	− 0.58	−1.77 - 0.61	0.342	–	–	–
	Neonatal mortality rate (per 1000 live births)	−2.27	−4.76 - 0.23	0.075	–	–	–
	Stunting (%)	0.47	−0.38 - 1.32	0.277	–	–	–

^a^Units of analyses are 192 (24 departments × 8 years). Variables in each group are adjusted for all other variables in the same group or above

For time- and confounder- adjusted regressions, conditional cash transfer JUNTOS and density of health professionals showed a significant and positive coefficient, while previous year expenditure and care seeking for pneumonia were also significant but negative, while urbanization fell only marginally short of statistical significance (Table 5).

In the final multilevel model, the random-effects component did not show statistical difference between departments.

## Discussion

Public RMNCH expenditure increased substantially over time in all departments of Peru, which is a remarkable achievement, and is unique within the universe of the Countdown country case studies. The magnitude of expenditure increase was lower for reproductive health, likely reflecting a lower political profile given to family planning [29], after concerns were raised regarding violation of reproductive rights during the implementation of the National Family Planning programme during the 1990s [30]. There was wide variation of expenditure across departments over time, but no consistent patterns across the RMNCH activities, that is we could not identify a defined profile of “best performers” or of departments lagging behind for each expenditure outcome.

Under the currently implemented results-based budgeting programmes aimed at mothers, newborns and children, funds are allocated according to each department’s performance [24]. However, we could not distinguish a consistent pattern where those departments with the highest increase in coverage of RMNCH interventions or with the highest decrease of stunting, neonatal or under-five mortality show the highest RMNCH expenditure. This may reflect the fact that RMNCH allocation and expenditure are not yet sufficiently focused on the departments with the worst indicators needing the greatest support [15, 31, 32], a drawback needing particular emphasis to guarantee sustainability of the RMNCH progress achieved by Peru.

Our ecological approach using hierarchical multilevel mixed-effects linear regression model did not show consistent influencing factors underlying public expenditure on RMNCH, except for previous year expenditure on reproductive health on maternal-neonatal health and on child health that showed a positive association with their corresponding expenditure variables. This finding may have various explanations.

First, the inertial nature of the allocation and expenditure, heavily based on historical financial allocation and expenditure, seems to have been still in place in Peru at sub-national level, at least until recently [15]. This may explain why expenditure was higher in departments with lower income inequality, lower coverage of conditional cash transfers and with lower levels of under-five health services utilization provided by the health insurance system (SIS). In brief, it may explain why the departments in most need did not seem to receive the highest priority. It may also explain that previous year expenditure influenced expenditure during the following year, most notably for reproductive and maternal-neonatal health.

Secondly, the results-based budgeting approach [24, 33, 34], which was scaled-up in Peru since 2009 as a salient characteristic of RMNCH and other crosscutting anti-poverty programmes, may need additional time to be reflected in clearly increased expenditure efficiency and effectiveness. This may be due to regional policy and system technical limitations for implementing effectively the interventions promoted by these programmes, and to low governance and accountability levels is still prominent, particularly at regional level. The results-based approach seeks to allocate budgets to different sectors at national and regional levels, based on the achievement of specific results in terms of implementation of interventions, coverage and impact indicators, such as the reduction of maternal, newborn and child mortality, and the reduction of stunting. Sound evidence about its impact on the quality of allocation and expenditure is still pending, particularly in developing countries [35, 36].

Third, decentralization is another process that was implemented in Peru with the promise of increased public efficiency [37]. However, their results so far have been mixed [38]. On one hand, it has resulted in significant budgetary transfers from the central to the regional governments, although key financial allocation decisions are still made centrally. On the other hand, it has not been accompanied by an effective development of regional and local technical capabilities and by efficient and transparent accountability mechanisms [39], hampering therefore a more adequate expenditure of financial resources by the regions [12]. Moreover, it seems to have reduced the executive capacity of the Ministry of Health in terms of the accomplishment of its public health functions [12].

Fourth, although we included in our analysis models a wide array of variables ranging from contextual factors to coverage of specific RMNCH interventions and child health impact indicators, they may have not fully captured the complex web of factors influencing decisions to allocate and to eventually spend on RMNCH. For instance, external and domestic factors perceived by politicians and policy makers as compelling enough to prioritize expenditure on RMNCH such as measures leading to fertility reduction seemed to have been present during the 1990s, but they became politically sensitive during the 2000s, partly due to concerns on human rights violations during the implementation of the Family Planning Programme in the 1990s [29].

Limited sample size related to coverage of individual RMNCH interventions may have also decreased the utility of our models, particularly coverage of vaccines and coverage of care seeking for pneumonia. To overcome this potential drawback we run a separate analysis with composite coverage index (CCI), which is a a weighted mean of the coverage of eight preventive and curative interventions different aspects of the continuum of care, including family planning, maternity care, child immunization, and case management [40]. We did not find significant influence of CCI on the RMNCH expenditure outcomes. Furthermore, sample size is carefully considered during the DHS standardisation process, excluding any variable having less than 50 households at departmental level.

Of note, we have performed previously a study on stunting reduction drivers in Peru at departmental level by using the same dataset and the same methodological hierarchical multilevel approach [41]. Although stunting is also a complex outcome driven by several factors, we were able to identify in our analyses several influencing distal, intermediate and proximal covariates.

Finally, our hierarchical mixed-effects model may be intrinsically limited to capture the whole set of complex factors influencing the expenditure on RMNCH. In this regard, we must underline that the random-effects component of the final adjusted regressions failed to show significant differences across departments, even if the expenditure trend over time was clearly positive for all departments. Thus, a mixed approach taking into account quantitative and qualitative approaches seems the best method to assess comprehensively factors underlying RMNCH expenditure in future studies. This may be particularly important at district level, where there is substantial heterogeneity between districts for various socioeconomic, political, cultural, and managerial factors. Inclusion of quality indicators is also warranted to better capture their influence on outcomes in hierarchical multilevel analyses, and fortunately this is the case in Peru, where recently quality indicators for antenatal care visits and birth attendances have been introduced in the DHS [42].

## Conclusions

In conclusion, our study shows that inertial public RMNCH expenditure is still predominant at departmental level, highlithing the need to further strengthen regional and local capabilities, to achieve more efficient budget execution at such levels. Although district level RMNCH expenditure in Peru has increased remarkably, the evidence on underlying factors influencing such increased expenditure warrants further research, most likely through a combination of quantitative and qualitative approaches, taking advantage of better and representative data locally collected.

## Additional files

Additional file 1: Per capita expenditure on reproductive health activities (constant US$ per woman of reproductive age), Peru: 2004–2012. (DOCX 19 kb)

Additional file 2: Per capita expenditure on maternal-neonatal health activities (constant 2012 US$ per pregnant woman), Peru: 2000–2012. (DOCX 18 kb)

Additional file 3: Per capita expenditure on child health activities (constant 2012 US$ per under-five child), Peru: 2000–2012. (DOCX 18 kb)

Additional file 4: Scatterplots of annual variation of expenditure indicators versus annual variation of selected RMNCH coverage and impact indicators. (DOCX 127 kb)

## Abbreviations

DHS: The Demographic and Health Surveys Program
ESSALUD: Social Security
GDP: Gross Domestic Product
MDG: Millennium Development Goals
RMNCH: Reproductive, maternal, neonatal and child health
SIS: Comprehensive Health Care Insurance
