# Supplementary material for: Understanding drivers of domestic public expenditure on reproductive, maternal, neonatal and child health in Peru at district level: an ecological study
Source: BMC Health Serv Res. 2018 Nov 6;18:833. doi: 10.1186/s12913-018-3649-x (PMC6219038; doi:10.1186/s12913-018-3649-x)
Supplement: Supplementary file 1 — Per capita expenditure on reproductive health activities (constant US$ per woman of reproductive age), Peru: 2004–2012. (DOCX 19 kb) [file 12913_2018_3649_MOESM1_ESM.docx]

**Additional file 1**

**Per capita expenditure on reproductive health activities (constant 2012 US$ per woman of reproductive age), Peru: 2004-2012**

| **Department** | **Per capita expenditure on reproductive health** | | | | | | | | |
| --- | --- | --- | --- | --- | --- | --- | --- | --- | --- |
|  | **2004** | **2005** | **2006** | **2007** | **2008** | **2009** | **2010** | **2011** | **2012** |
| **Amazonas** | 0.91 | 0.97 | 1.01 | 1.07 | 1.18 | 1.19 | 0.75 | 3.29 | 5.02 |
| **Ancash** | 0.75 | 0.85 | 0.92 | 1.06 | 1.27 | 1.36 | 1.68 | 3.91 | 7.81 |
| **Apurimac** | 0.63 | 1.00 | 1.49 | 2.36 | 3.84 | 6.07 | 4.39 | 9.00 | 14.70 |
| **Arequipa** | 0.22 | 0.30 | 0.35 | 0.48 | 0.69 | 0.86 | 1.51 | 3.65 | 10.64 |
| **Ayacucho** | 0.77 | 1.26 | 1.59 | 2.61 | 4.42 | 7.32 | 5.51 | 9.27 | 9.50 |
| **Cajamarca** | 0.13 | 0.13 | 0.30 | 0.77 | 2.04 | 4.92 | 2.30 | 4.39 | 6.13 |
| **Cusco** | 0.45 | 0.41 | 0.35 | 0.32 | 0.31 | 1.50 | 1.11 | 3.68 | 5.89 |
| **Huancavelica** | 0.35 | 0.25 | 2.98 | 0.44 | 1.40 | 4.04 | 2.46 | 4.03 | 5.56 |
| **Huanuco** | 0.36 | 0.53 | 0.80 | 1.19 | 1.81 | 2.41 | 1.44 | 2.99 | 5.01 |
| **Ica** | 0.10 | 0.18 | 0.28 | 0.50 | 0.89 | 1.57 | 0.76 | 3.31 | 9.72 |
| **Junin** | 0.32 | 0.48 | 0.72 | 1.10 | 1.75 | 2.41 | 0.52 | 2.70 | 4.50 |
| **La Libertad** | 0.90 | 0.84 | 0.96 | 1.22 | 1.60 | 1.90 | 1.38 | 3.59 | 5.90 |
| **Lambayeque** | 0.02 | 0.00 | 0.01 | 0.05 | 0.37 | 2.35 | 2.32 | 4.68 | 7.95 |
| **Lima** | 2.17 | 2.18 | 2.06 | 2.13 | 2.26 | 2.19 | 3.06 | 2.67 | 4.80 |
| **Loreto** | 0.87 | 1.02 | 1.08 | 1.26 | 1.52 | 1.67 | 1.38 | 5.19 | 9.72 |
| **Madre de Dios** | 0.44 | 0.78 | 1.27 | 2.23 | 4.06 | 6.72 | 5.76 | 11.16 | 32.29 |
| **Moquegua** | 0.17 | 0.19 | 0.22 | 0.26 | 0.32 | 0.37 | 0.37 | 2.58 | 5.91 |
| **Pasco** | 0.40 | 0.58 | 0.75 | 1.08 | 1.61 | 2.31 | 1.79 | 6.54 | 6.43 |
| **Piura** | 0.64 | 0.75 | 0.81 | 0.96 | 1.18 | 1.29 | 0.95 | 2.97 | 2.89 |
| **Puno** | 1.26 | 1.38 | 1.36 | 1.51 | 1.72 | 1.76 | 1.48 | 3.98 | 5.80 |
| **San Martin** | 1.29 | 1.82 | 2.59 | 3.66 | 5.35 | 7.49 | 1.61 | 6.54 | 12.02 |
| **Tacna** | 0.09 | 0.13 | 0.18 | 0.25 | 0.36 | 0.50 | 0.35 | 3.07 | 3.63 |
| **Tumbes** | 0.92 | 0.95 | 0.96 | 1.00 | 1.07 | 1.04 | 0.56 | 2.76 | 7.73 |
| **Ucayali** | 0.98 | 1.00 | 1.03 | 1.05 | 1.11 | 1.06 | 0.69 | 4.16 | 6.84 |
