# Supplementary material for: Understanding drivers of domestic public expenditure on reproductive, maternal, neonatal and child health in Peru at district level: an ecological study
Source: BMC Health Serv Res. 2018 Nov 6;18:833. doi: 10.1186/s12913-018-3649-x (PMC6219038; doi:10.1186/s12913-018-3649-x)
Supplement: Supplementary file 2 — Per capita expenditure on maternal-neonatal health activities (constant 2012 US$ per pregnant woman), Peru: 2000–2012. (DOCX 18 kb) [file 12913_2018_3649_MOESM2_ESM.docx]

**Additional file 2**

**Per capita expenditure on maternal-neonatal health activities (constant 2012 US$ per pregnant woman), Peru: 2000-2012**

| **Department** | **Per capita expenditure on maternal-neonatal health** | | | | | | | | | |
| --- | --- | --- | --- | --- | --- | --- | --- | --- | --- | --- |
|  | **2000** | **2004** | **2005** | **2006** | **2007** | **2008** | **2009** | **2010** | **2011** | **2012** |
| **Amazonas** | 26.04 | 290.21 | 334.51 | 324.15 | 353.36 | 87.71 | 233.23 | 134.99 | 305.21 | 435.69 |
| **Ancash** | 24.37 | 136.84 | 140.75 | 103.35 | 127.54 | 93.37 | 121.14 | 95.33 | 276.85 | 430.09 |
| **Apurimac** | 43.67 | 252.20 | 350.13 | 359.90 | 341.73 | 340.07 | 376.75 | 371.00 | 529.52 | 917.52 |
| **Arequipa** | 13.79 | 237.08 | 210.18 | 196.98 | 227.48 | 208.44 | 372.79 | 378.51 | 485.90 | 499.28 |
| **Ayacucho** | 38.55 | 329.73 | 408.64 | 360.32 | 301.89 | 403.97 | 461.54 | 521.64 | 530.71 | 815.56 |
| **Cajamarca** | 32.53 | 205.05 | 261.22 | 247.67 | 284.73 | 128.79 | 246.35 | 208.19 | 293.75 | 475.07 |
| **Cusco** | 22.34 | 170.27 | 186.71 | 224.31 | 220.71 | 192.08 | 248.07 | 254.37 | 321.94 | 496.02 |
| **Huancavelica** | 21.56 | 92.17 | 251.49 | 213.63 | 154.97 | 125.46 | 144.19 | 163.86 | 184.86 | 334.46 |
| **Huanuco** | 16.51 | 130.44 | 320.43 | 315.47 | 361.29 | 188.68 | 206.08 | 171.70 | 218.63 | 338.34 |
| **Ica** | 9.40 | 152.07 | 143.42 | 163.81 | 119.26 | 86.34 | 75.47 | 73.93 | 105.69 | 500.09 |
| **Junin** | 24.77 | 35.30 | 169.15 | 320.65 | 181.27 | 137.40 | 223.74 | 110.90 | 207.42 | 336.69 |
| **La Libertad** | 11.10 | 216.16 | 181.22 | 156.80 | 66.97 | 222.35 | 182.25 | 214.04 | 305.58 | 443.63 |
| **Lambayeque** | 18.93 | 170.53 | 244.14 | 260.73 | 334.64 | 192.37 | 228.56 | 187.11 | 287.20 | 423.54 |
| **Lima** | 61.58 | 147.49 | 154.67 | 137.00 | 132.34 | 295.22 | 356.34 | 372.40 | 358.19 | 641.01 |
| **Loreto** | 20.38 | 200.76 | 293.97 | 304.29 | 386.29 | 107.92 | 138.23 | 122.49 | 380.25 | 418.20 |
| **Madre de Dios** | 22.59 | 214.19 | 209.83 | 183.71 | 154.35 | 303.11 | 394.91 | 349.27 | 455.04 | 714.64 |
| **Moquegua** | 113.83 | 292.13 | 264.60 | 201.09 | 197.90 | 353.24 | 236.57 | 85.24 | 629.31 | 1093.36 |
| **Pasco** | 9.99 | 283.83 | 348.12 | 260.37 | 235.62 | 109.58 | 171.88 | 127.13 | 253.82 | 311.97 |
| **Piura** | 8.89 | 204.50 | 194.67 | 189.86 | 187.43 | 131.59 | 151.05 | 137.51 | 235.39 | 353.63 |
| **Puno** | 28.50 | 44.20 | 126.48 | 131.81 | 146.14 | 143.37 | 151.89 | 131.36 | 215.20 | 296.38 |
| **San Martin** | 53.40 | 35.43 | 181.50 | 243.21 | 254.45 | 239.96 | 327.93 | 258.43 | 312.60 | 376.89 |
| **Tacna** | 161.74 | 102.84 | 228.23 | 232.53 | 212.06 | 596.22 | 598.30 | 662.80 | 764.02 | 977.13 |
| **Tumbes** | 11.50 | 40.31 | 275.08 | 287.72 | 264.47 | 76.48 | 79.74 | 85.68 | 553.89 | 976.94 |
| **Ucayali** | 30.58 | 70.69 | 205.74 | 210.22 | 217.61 | 118.53 | 157.08 | 118.67 | 362.22 | 559.12 |
