# Supplementary material for: Understanding drivers of domestic public expenditure on reproductive, maternal, neonatal and child health in Peru at district level: an ecological study
Source: BMC Health Serv Res. 2018 Nov 6;18:833. doi: 10.1186/s12913-018-3649-x (PMC6219038; doi:10.1186/s12913-018-3649-x)
Supplement: Supplementary file 3 — Per capita expenditure on child health activities (constant 2012 US$ per under-five child), Peru: 2000–2012. (DOCX 18 kb) [file 12913_2018_3649_MOESM3_ESM.docx]

**Additional file 3**

**Per capita expenditure on child health activities (constant 2012 US$ per under-five child), Peru: 2000-2012**

| **Department** | **Per capita expenditure on child health** | | | | | | | | | |
| --- | --- | --- | --- | --- | --- | --- | --- | --- | --- | --- |
|  | **2000** | **2004** | **2005** | **2006** | **2007** | **2008** | **2009** | **2010** | **2011** | **2012** |
| **Amazonas** | 8.23 | 37.44 | 37.87 | 54.06 | 60.52 | 200.81 | 49.03 | 142.92 | 146.03 | 212.67 |
| **Ancash** | 4.46 | 6.53 | 5.77 | 12.03 | 15.94 | 187.91 | 23.40 | 84.42 | 102.72 | 151.05 |
| **Apurimac** | 2.66 | 18.14 | 45.22 | 27.69 | 44.50 | 420.86 | 70.90 | 200.91 | 223.37 | 290.07 |
| **Arequipa** | 10.56 | 10.99 | 24.15 | 30.65 | 36.54 | 70.55 | 51.15 | 103.48 | 93.29 | 114.38 |
| **Ayacucho** | 3.85 | 14.50 | 15.49 | 21.06 | 58.40 | 356.53 | 86.53 | 202.28 | 200.09 | 259.72 |
| **Cajamarca** | 3.46 | 11.88 | 13.47 | 19.78 | 24.37 | 275.07 | 62.11 | 114.12 | 126.01 | 169.67 |
| **Cusco** | 6.50 | 13.26 | 14.55 | 23.37 | 29.70 | 221.70 | 34.95 | 100.38 | 101.21 | 143.51 |
| **Huancavelica** | 7.88 | 8.19 | 12.22 | 16.68 | 17.12 | 359.53 | 29.35 | 134.62 | 138.62 | 221.04 |
| **Huanuco** | 2.50 | 46.67 | 15.52 | 24.68 | 30.48 | 350.34 | 40.19 | 97.47 | 86.21 | 161.01 |
| **Ica** | 0.91 | 8.90 | 5.49 | 8.46 | 8.71 | 27.44 | 12.50 | 65.23 | 46.50 | 181.16 |
| **Junin** | 4.32 | 51.84 | 8.37 | 19.05 | 9.28 | 112.60 | 31.58 | 79.86 | 98.55 | 128.49 |
| **La Libertad** | 1.91 | 7.52 | 9.08 | 18.85 | 22.18 | 181.07 | 32.20 | 89.26 | 86.25 | 116.93 |
| **Lambayeque** | 5.06 | 14.62 | 12.36 | 19.59 | 27.87 | 88.30 | 42.72 | 88.10 | 76.67 | 98.53 |
| **Lima** | 15.99 | 41.57 | 56.54 | 48.00 | 85.93 | 77.29 | 117.48 | 97.30 | 74.58 | 124.04 |
| **Loreto** | 4.24 | 15.00 | 16.41 | 24.56 | 32.96 | 90.03 | 28.35 | 100.41 | 114.29 | 145.35 |
| **Madre de Dios** | 8.78 | 20.05 | 7.55 | 25.47 | 30.24 | 111.91 | 364.35 | 120.84 | 180.51 | 257.05 |
| **Moquegua** | 4.71 | 14.29 | 8.57 | 12.44 | 56.67 | 121.37 | 88.37 | 57.16 | 226.50 | 447.89 |
| **Pasco** | 3.27 | 14.98 | 12.77 | 17.09 | 18.44 | 103.72 | 33.01 | 101.31 | 138.69 | 184.05 |
| **Piura** | 8.56 | 11.41 | 11.10 | 17.67 | 20.87 | 106.56 | 22.80 | 74.11 | 123.08 | 134.08 |
| **Puno** | 7.90 | 38.44 | 7.70 | 10.54 | 14.30 | 160.88 | 21.59 | 86.55 | 89.29 | 139.18 |
| **San Martin** | 3.71 | 51.68 | 14.17 | 20.48 | 23.57 | 126.37 | 66.18 | 98.10 | 98.11 | 143.96 |
| **Tacna** | 2.76 | 8.96 | 7.90 | 14.85 | 16.06 | 110.30 | 98.72 | 171.23 | 182.00 | 225.96 |
| **Tumbes** | 9.97 | 80.56 | 23.91 | 31.61 | 33.92 | 68.12 | 11.01 | 46.15 | 130.48 | 271.24 |
| **Ucayali** | 7.71 | 45.78 | 19.23 | 21.24 | 28.88 | 128.35 | 31.01 | 82.42 | 137.84 | 189.03 |
