# Supplementary material for: Understanding drivers of domestic public expenditure on reproductive, maternal, neonatal and child health in Peru at district level: an ecological study
Source: BMC Health Serv Res. 2018 Nov 6;18:833. doi: 10.1186/s12913-018-3649-x (PMC6219038; doi:10.1186/s12913-018-3649-x)
Supplement: Supplementary file 4 — Scatterplots of annual variation of expenditure indicators versus annual variation of selected RMNCH coverage and impact indicators. (DOCX 127 kb) [file 12913_2018_3649_MOESM4_ESM.docx]

**Additional file 4**

**Scatterplots of annual variation of expenditure indicators versus annual variation of selected RMNCH coverage and impact indicators.**

**
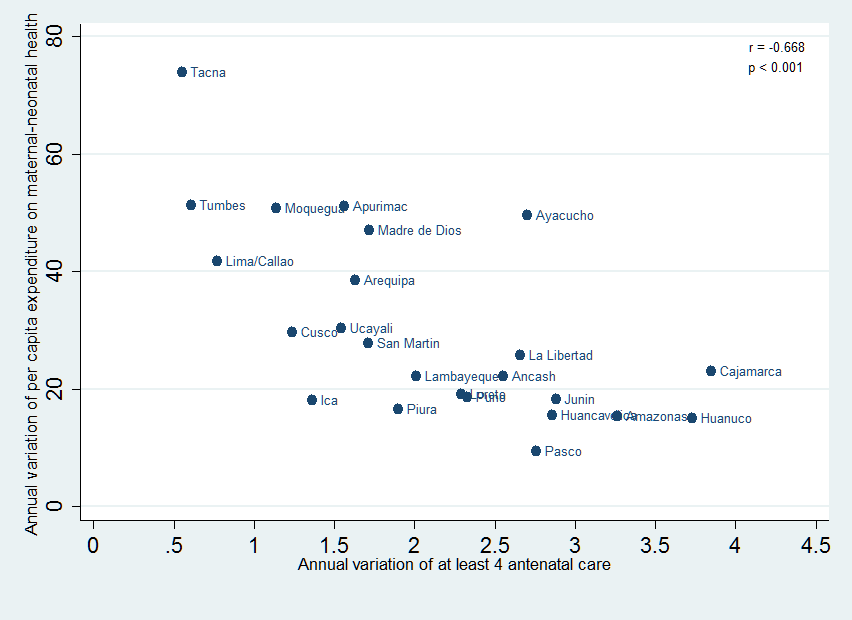
**

**
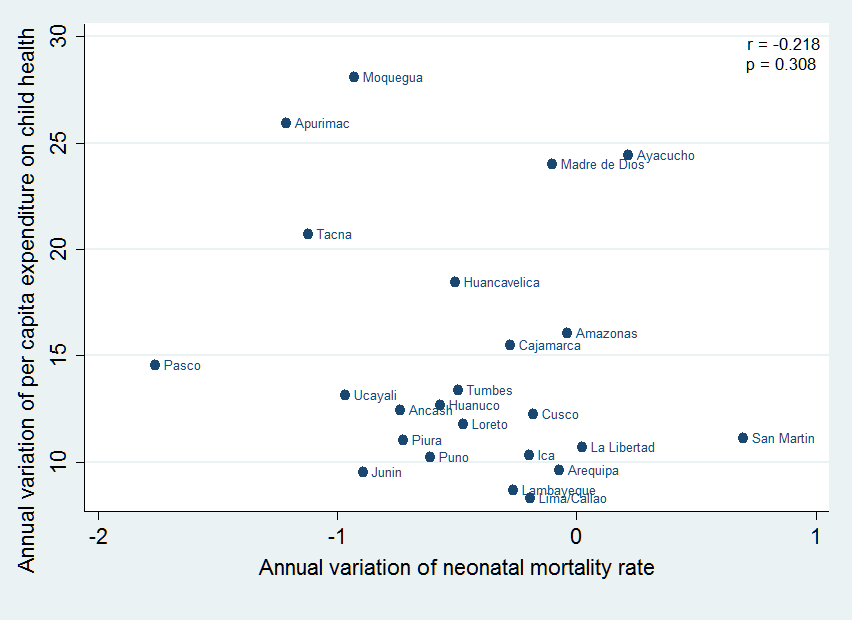
**
